# Linaclotide-induced electrolyte secretion in human and rat colon: Ussing chamber studies

**DOI:** 10.1186/2050-6511-16-S1-A94

**Published:** 2015-09-02

**Authors:** Boris Tchernychev, Gerhard Hannig, David Arthur, Pei Ge, Jenny Tobin, Inmaculada Silos-Santiago

**Affiliations:** 1 Ironwood Pharmaceuticals, Cambridge, MA 02142, USA; 2 Biopta Ltd., Glasgow, G61 1QH, UK

## Background

Linaclotide is a guanylyl cyclase C (GC-C) agonist approved for treatment of adult patients with irritable bowel syndrome with constipation (IBS-C). Linaclotide binding to the extracellular receptor domain of GC-C activates the intracellular catalytic domain of GC-C, resulting in the generation of cyclic GMP (cGMP). cGMP, via a pathway leading to activation of the cystic fibrosis transmembrane conductance regulator (CFTR) ion channel, stimulates Cl- and HCO3-secretion and concomitant inhibition of Na+ absorption by sodium/hydrogen exchanger 3 (NHE3), driving the efflux of water into the lumen and accelerating bowel transit. However, the effect of linaclotide on electrolyte transport in the lower gastrointestinal tract has not yet been characterized. This study specifically examined the effect of linaclotide on electrolyte transport in the colon. We used an *in vitro* Ussing chamber assay to measure the secretion of electrolytes in human ascending colon segments and in rat proximal colon in response to linaclotide treatment.

## Materials and methods

Colonic mucosa was isolated using either sharp (human) or blunt (rat) dissection and ion transport was monitored by measuring short-circuit current (Isc).

## Results

Stimulation with linaclotide elicited a concentration-dependent short-circuit current across rat colonic epithelium. The Isc response reached its maximum at 100 nM of linaclotide with an EC50 of 9.8 nM in rat colonic mucosa. Linaclotide at concentrations of 30 nM and 1000 nM added to human colonic mucosa also induced a trans-epithelial current. As the CFTR ion channel and Na-K-Cl co-transporter NKCC1 are well-characterized transporters regulating the flux of Cl-anions across the intestinal epithelium, we further studied the effects of NKCC1 and CFTR antagonists on GC-C–mediated short-circuit current. Addition of NKCC1 inhibitor bumetanide (100 μM) to the basolateral bath significantly inhibited linaclotide-induced transepithelial current in human colonic mucosa. In contrast, bumetanide had no effect on linaclotide-induced transepithelial current in rat colonic mucosa, suggesting that in the rat proximal colon, flux of Cl-anions is mediated by alternate transporters. Preincubation of rat colonic mucosa with the CFTR antagonist GlyH-101 attenuated the sustained increase in Isc observed in response to treatment with 100 nM linaclotide. The effect of GlyH-101 on Isc inhibition was more profound after the colonic epithelium was stimulated with a sub-EC50 (6 nM) concentration of linaclotide.

## Conclusions

We have demonstrated that linaclotide treatment induces a transepithelial ion current in the human ascending colon and rat proximal colon. This ion current is in part driven by Cl-anion secretion into the intestinal lumen.

